# KDS2010, a Newly Developed Reversible MAO-B Inhibitor, as an Effective Therapeutic Candidate for Parkinson’s Disease

**DOI:** 10.1007/s13311-021-01097-4

**Published:** 2021-10-05

**Authors:** Min-Ho Nam, Jong-Hyun Park, Hyo Jung Song, Ji Won Choi, Siwon Kim, Bo Ko Jang, Hyung Ho Yoon, Jun Young Heo, Hyowon Lee, Heeyoung An, Hyeon Jeong Kim, Sun Jun Park, Doo-Wan Cho, Young-Su Yang, Su-Cheol Han, Sangwook Kim, Soo-Jin Oh, Sang Ryong Jeon, Ki Duk Park, C. Justin Lee

**Affiliations:** 1 Center for Neuroscience, Brain Science Institute, Korea Institute of Science and Technology (KIST), Seoul, 02792 Republic of Korea; 2 Department of KHU-KIST Convergence Science and Technology, Kyung Hee University, Seoul, 02453 Korea; 3 Convergence Research Center for Diagnosis, Treatment and Care System of Dementia, KIST, Seoul, 02792 Republic of Korea; 4 Division of Bio-Med Science & Technology, KIST School, Korea University of Science and Technology, Seoul, 02792 Republic of Korea; 5 Department of Neurological Surgery, Asan Medical Center, University of Ulsan College of Medicine, Seoul, 05505 Korea; 6 Department of Medical Science, College of Medicine, Chungnam National University, Daejeon, 35015 Republic of Korea; 7 Center for Cognition and Sociality, Institute for Basic Science, Daejeon, 34126 Republic of Korea; 8 Department of Biotechnology, Yonsei University, Seoul, 03722 Republic of Korea; 9 Jeonbuk Branch Institute, Korea Institute of Toxicology, Jeonbuk, 56212 Republic of Korea; 10 Neurobiogen Co., LTD, Seocho-gu, Seoul 9 Republic of Korea

**Keywords:** MAO-B inhibitor, Parkinson’s disease, Pharmacology, α-Aminoamide derivative, Reactive glia

## Abstract

Monoamine oxidase-B (MAO-B) is a well-established therapeutic target for Parkinson’s disease (PD); however, previous clinical studies on currently available irreversible MAO-B inhibitors have yielded disappointing neuroprotective effects. Here, we tested the therapeutic potential of KDS2010, a recently synthesized potent, selective, and reversible MAO-B inhibitor in multiple animal models of PD. We designed and synthesized a series of α-aminoamide derivatives and found that derivative KDS2010 exhibited the highest potency, specificity, reversibility, and bioavailability (> 100%). In addition, KDS2010 demonstrated significant neuroprotective and anti-neuroinflammatory efficacy against nigrostriatal pathway destruction in the mouse MPTP model of parkinsonism. Treatment with KDS2010 also alleviated parkinsonian motor dysfunction in 6-hydroxydopamine-induced and A53T mutant α-synuclein overexpression rat models of PD. Moreover, KDS2010 showed virtually no toxicity or side effects in non-human primates. KDS2010 could be a next-generation therapeutic candidate for PD.

**Supplementary Information:**

The online version contains supplementary material available at 10.1007/s13311-021-01097-4.

## Introduction

Parkinson’s disease (PD) is a neurodegenerative disorder primarily affecting the nigrostriatal dopaminergic pathway and manifesting with prominent motor deficits such as rigidity, bradykinesia, resting tremor, and gait disturbance [1]. Various drugs have been developed and prescribed for alleviating these motor symptoms by enhancing dopaminergic transmission, including the dopamine precursor L-Dopa [2], dopamine receptor agonists [3], catechol-*O*-methyl transferase inhibitors [4], and monoamine oxidase-B (MAO-B) inhibitors [5].

Monoamine oxidase-B, an enzyme located in the outer mitochondrial membrane of astrocytes, catalyzes the oxidative deamination of biogenic amines, including dopamine [6]. Therefore, MAO-B inhibitors are believed to exert anti-parkinsonian effects by blocking the degradation of dopamine [7]. However, recent studies have demonstrated that MAO-B expression is dramatically elevated in reactive astrocytes and that MAO-B is responsible for astrocytic GABA synthesis through the putrescine degradation pathway [8–11]. Moreover, a recent study revealed that GABA from reactive astrocytes in the substantia nigra pars compacta (SNpc) tonically inhibits neighboring dopaminergic (DAergic) neurons, thereby suppressing the synthesis and release of dopamine [8]. These findings suggest that excessive MAO-B-mediated astrocytic GABA synthesis is critically involved in PD pathology and that MAO-B inhibitors may reduce PD symptoms by suppressing astrocytic GABA synthesis and disinhibiting nigral DAergic neurons.

Irreversible MAO-B inhibitors, such as selegiline and rasagiline, are widely prescribed to PD patients. While these agents are effective in delaying the need for dopamine replacement therapy with levodopa and induce relatively few adverse effects, several studies have suggested little actual neuroprotective potential [12, 13]. In a mouse model of Alzheimer’s disease (AD), this limited neuroprotective effect of long-term irreversible MAO-B inhibitor treatment was attributed to short-lived action due to a rebound in astrocytic GABA accumulation through a diamine oxidase (DAO)-mediated compensatory mechanism [9, 14]. To overcome this limitation of irreversible MAO-B inhibitors, a reversible MAO-B inhibitor, safinamide, was developed as a PD drug. However, safinamide has several additional actions including blockade of voltage-gated sodium and calcium channels, which could contribute to adverse events [15]. Based on the known structure and molecular interactions of available MAO-B inhibitors, we synthesized a series of α-aminoamine derivatives, including one with a biphenyl moiety, KDS2010, and demonstrated that this compound is a potent, highly selective, and reversible MAO-B inhibitor [14]. Further, we reported that KDS2010 rescued memory deficits in a mouse model of AD [14]. In the current study, we screened additional α-aminoamide derivatives for more potent and selective MAO-B inhibitors and tested the therapeutic potential of KDS2010 in several animal models of PD. Furthermore, we studied the pharmacokinetics and toxicity of KDS2010 in non-human primates to validate its safety as a clinical candidate drug.

## Methods

### Chemical Synthesis

Compound synthesis and analysis methods are described in Supplemental Information.

### In Vitro Monoamine Oxidase-A (MAO-A) and Monoamine Oxidase-B Enzyme Assays

Analyses of the 50% inhibitory concentration (IC_50_) for in vitro MAO-A and MAO-B enzyme activities were performed as described previously [16]. In brief, human recombinant MAO-A (hMAO-A) and MAO-B (hMAO-B) (Sigma Aldrich) were diluted in 50 mM phosphate buffer (~ 0.3 μg MAO-A protein/well or ~ 2.5 μg MAO-B protein/well), and the test compound was added in DMSO to a final concentration from 0.1 nM to 10 μM. The amount of hydrogen peroxide (H_2_O_2_) released after addition of enzyme substrate (*p*-tyramine for MAO-A or benzylamine for MAO-B) was quantified by measuring the absorbance increase at 570 nm on a microplate reader.

### H_2_O_2_ Scavenging Capacity Test

To determine the H_2_O_2_ scavenging capacity of the test compound, H_2_O_2_ concentration was measured using the modified Amplex red/horseradish peroxidase (HRP) detection method as described previously [14]. Briefly, a H_2_O_2_ solution was incubated with the test compound for 10 min at room temperature (RT), and then the reaction solution containing 20 mM Amplex red and 200 U/mL HRP was added. The absorbance was measured at 570 nm using a microplate reader. The H_2_O_2_ scavenging capacities were compared to a blank negative control and to the known H_2_O_2_ scavenger epigallocatechin gallate (EGCG) as a positive control.

### Animals

All mice and rats were housed in a temperature- and humidity-controlled environment under a 12-h light/12-h dark cycle with free access to food and water. Animal handling and care were performed according to the directives of the Animal Care and Use Committee of the Korea Institute of Science and Technology (KIST) (Seoul, Korea). Animal studies were performed in compliance with Animal Research: Reporting of In Vivo Experiments (ARRIVE) guidelines [17].

### Disease Modeling and Treatment

Mice and rats were randomly allocated to individual treatment groups receiving the indicated disease-modeling agent (e.g., MPTP, 6-hydroxydopamine, or A53T-α-synuclein overexpression virus) and candidate treatment agent (KDS2010, selegiline, or safinamide) or appropriate vehicle control. Group allocation and outcome assessment, but not the conduct of experiments and data analysis, were performed in a blinded manner. Throughout the study, confounders were not controlled. An acclimation period of 7 days was allowed for animals to stabilize in a new environment before model induction. Following disease modeling (described below), mice and rats were treated with 10 mg/kg per day KDS2010, selegiline, safinamide, or vehicle by oral administration. Treatment drugs were dissolved in 100 μL of water and total dose (in mg) was calculated according to individual body weight (in kg). In total, 131 mice and 46 rats were treated, tested, and sacrificed for subsequent histopathology. An experiment was halted if body weight decreased by 20% or more after MPTP or 6-hydroxydopamine (6-OHDA) treatment.

The MPTP model was established using the acute regimen of four intraperitoneal (*i.p.*) injections of MPTP-HCl (M0896, Sigma Aldrich, 2 mg/mL in saline, 20 mg/kg for one injection) at 2-h intervals. All MPTP use and safety precautions were strictly followed as described [18]. The A53T mutant α-synuclein model was established as previously described [8]. Under chloral hydrate general anesthesia, all rats received unilateral injections of 2 μL AAV-CMV-A53T-Asyn or AAV-CMV-EGFP viral solution (5.46 × 10^13^ GC/mL, packaged by the KIST virus facility) into the right SNpc (AP − 5.3 mm, ML − 2.3 mm, DV − 7.6 mm relative to the bregma; 0.2 μL/min) [19, 20]. In total, 1.92 × 10^11^ GC of AAV-A53T or AAV-CMV-EGFP viruses was injected into each SNpc. The A53T model rats started KDS2010 treatment 3 weeks after virus injection. Finally, the 6-OHDA model was prepared as previously described [21]. Under general anesthesia, all rats received unilateral injections of 8 μg 6-OHDA (Sigma Aldrich) in 4 μL saline/0.1% ascorbic acid into the right medial forebrain bundle (AP − 2.2 mm and L + 1.5 mm relative to the bregma, V − 8.0 mm from the dura) [19], with the tooth bar set at + 4.5 mm. To confirm successful modeling (6-OHDA-induced neurodegeneration), rotation induced by 0.25 mg/kg subcutaneous (*s.c.*) apomorphine (Sigma Aldrich) was measured using an automated Rotameter (Panlab, Barcelona, Spain), with 6 rpm considered sufficient for subsequent treatment testing. Based on a previous report showing that 3 μg 6-OHDA was sufficient to denervate dopaminergic fibers within 3 weeks [22] and preliminary observations indicating that the treatment regimen employed here results in even more severe denervation, KDS2010 treatment was started ~ 17 days after 6-OHDA injection.

### MAO-B Assay in Brain Tissues

The MAO-B enzyme activity in brain tissues from 6-OHDA model rats was measured using a commercially available kit (A12214, Thermo Fisher Scientific) following the manufacturer’s protocol. First, the SNpc mitochondrial fraction was isolated as follows. Tissue was homogenized in buffer A (250 mM sucrose, 2 mM HEPES [pH 7.4], 0.1 mM EGTA) and centrifuged at 571 × *g* for 10 min. The supernatant was then centrifuged at 14,290 × *g* for 10 min, and the pellet re-suspended in buffer B solution (25 mM potassium phosphate, 5 mM MgCl_2_). The suspension was centrifuged at 15,339 × *g* for 10 min and the pellet containing mitochondria re-suspended in reaction buffer (0.05 M sodium phosphate). Total protein concentration was determined using a bicinchoninic acid (BCA) protein assay kit (#23228 and #23224, Thermo Scientific) and MAO-B activity measured by fluorometric assay. The reaction was initiated by adding 100 μL of a reaction mixture containing Amplex red reagent (400 μM), HRP (2 U/mL), and the specific MAO-B substrate benzylamine (2 mM) to each mitochondrial sample in multiwell plates. Plates were incubated for 30 min at 37°C under darkness and the absorbance monitored at 570 nm using a microplate reader (Infinite M 200 Pro, Tecan). Reaction buffer alone was used as a negative control and H_2_O_2_ (10 μM) as a positive control.

### DAB Staining

Coronal sections for striatum and SNpc were prepared at 30-μm thickness and immunostained using a DAB staining kit (TL-060-QHD, Thermo, MA, USA). Briefly, the sections were incubated in Hydrogen Peroxide Block (TA-060-HP, Thermo) for 10 min, washed three times in phosphate-buffered saline (PBS), incubated for 5 min in Ultravision Block (TA-060-UB, Thermo), washed three times in PBS, and then immunostained overnight at 4°C on a shaker with rabbit anti-tyrosine hydroxylase (TH) (Pel-freez, p40101-0, 1:500) or rabbit anti-Iba1 (Wako, 019–19741, 1:500) in a blocking solution of 0.1 M PBS containing 0.3% Triton-X and 2% ready-to-use donkey serum (GTX30972, Genetex, CA, USA). After washing in PBS 3 times, sections were incubated in Primary Antibody Amplifier Quanto (TA-060-QPB, Thermo) for 5 min, washed again in PBS, incubated in HRP Polymer Quanto for 1 h, washed 4 times in PBS, and dipped for 30 s in a 1:10 mixture of DAB + chromogen and DAB + substrate buffer (K3468, Dako, Denmark). Finally, sections were washed, immersed in mounting medium, and dried.

An unbiased stereological estimation of total TH-positive neuronal number in the SN area was performed using Stereo Investigator 11 (11.01.2 64-bit, MBF Bioscience). Every 6th section including the SN was stained with anti-TH and the number of TH-positive neurons counted under low magnification (× 10) from the rostral tip of the SNpc to the caudal end of the substantia nigra pars reticulate (SNpr). Briefly, an unbiased counting frame of known area (47.87 × 36.19 µm = 1,733 µm^2^) was placed randomly on the first counting area and systematically moved through all counting areas until the entire delineated area was sampled. The total number of neurons per section was estimated according to the optical fractionator formula. The total number of SNpc neurons in a hemisphere was then obtained by multiplying the number counted using Stereo Investigator 11 by the slice interval (six).

### Nissl Staining

Other 30-μm-thick coronal sections from the SNpc were Nissl stained to assess viable cell number. Briefly, slices were stained with 0.1% cresyl violet solution for 3 min, rinsed with distilled water, and placed in a chamber filled with 70% ethanol for 1 min. For differentiation of staining, slices were incubated in 95% ethanol for 5 min, then dehydrated with 100% ethanol for 5 min, cleared by washing twice in xylene, and mounted with permanent mounting medium. A series of bright field images were obtained using an Olympus microscope. The slices were classified according to AP coordinates and Nissl-positive neurons counted using the counting tool of ImageJ (NIH, MD, USA).

### Slice Immunostaining and Image Quantification

Sections were first incubated for 1 h in blocking solution (0.3% Triton-X and 2% normal serum in 0.1 M PBS) and then immunostained with a mixture of rabbit anti-TH (Pel-freez, p40101-0, 1:500) and chicken anti-glial fibrillary acidic protein (GFAP) (Millipore, AB5541, 1:500) in blocking solution at 4°C. After extensive washing, sections were incubated with corresponding fluorescent secondary antibodies for 2 h and then washed with three times in PBS. Finally, sections were mounted with fluorescent mounting medium (S3023, Dako) and dried. A series of fluorescent images were obtained using an A1 Nikon confocal microscope, and Z-stack images in 3-μm steps were processed for further analysis using ImageJ. Any alteration in brightness or contrast was applied equally to the entire image set. Immunospecificity was confirmed by omitting the primary antibody or by changing the fluorescent probes of the secondary antibodies.

For quantifying GFAP intensity as a measure of astroglial reactivity, we first defined the SNpc as the ROI based on TH immunostaining (under 10 × magnification) and digitized images at 8-bit using ImageJ. For quantifying Iba1-positive cell density, we manually counted the number of Iba1-positive cells in a 40 × image field and divided it by the area. The GFAP-positive volume and LCN2 intensity in GFAP-positive cells were measured using Imaris 8.2. Briefly, image stacks of GFAP-positive astrocytes and Iba1-positive microglial cells were rendered in 3D using Imaris 8.2. Each separate surface was considered an ROI and the LCN2 intensity in each ROI was measured.

### Western Blotting

Samples of the SNpc were homogenized, lysed with 1 × RIPA buffer (MB-030–0050, ROCKLAND; #320103, bio masher II, Nippi), and centrifuged at 13,000 × *g* for 10 min to obtain the soluble protein fraction. Soluble proteins were separated by sodium dodecyl sulfate–polyacrylamide gel electrophoresis (SDS-PAGE, #4561083, Bio-Rad) at 25 μg per gel lane and transferred to polyvinylidene difluoride membranes (IB24001, Thermo Fisher). The membranes were blocked with 3% bovine serum albumin in 0.1%Tris-buffered saline (20 mM Tris–HCl [pH 7.5] and 150 mM NaCl) supplemented with 0.1% Tween-20 (TBST) at RT for 1 h and then incubated at 4°C overnight in the same blocking solution containing rabbit anti-TH (1:1,000, P40101-150, Pel-Freez), rabbit anti-iNOS/NOS Type II (1:1,000, #610332, BD Biosciences), rabbit anti-β-actin (1:1,000, ab8227, Abcam), and chicken anti-GFAP (AB5541, Millipore). Membranes were washed with 0.1% TBST and then incubated at RT for 2 h with anti-rabbit IgG-peroxidase (1:2000, NIF824, Amersham) and HRP-conjugated rabbit anti-chicken IgG (1:2,000, AP162P, Millipore) in TBST containing 5% skim milk power. Antigen–antibody complexes were visualized using Pierce ECL western blotting substrate (#1705061, Bio-Rad).

### Behavioral Tests

Motor deficits of the MPTP-induced PD mouse model were assessed using the vertical grid test as described in the previous study [23]. Briefly, after 2 days of habituation to the testing apparatus, individual mice were gently placed 3 cm from the top, facing upward, and allowed to turn around and climb down. We also performed a coat hanger test as described [24–26]. Briefly, a mouse was gently hung on the middle of a coat hanger and allowed to ascend to the top. Performance was graded as follows: 0, falling off the hanger within 20 s; 1, moving to the side-bar of the hanger; 2, reaching the side-bar of the hanger; 3, climbing the side-bar of the hanger; 4, moving to the top of the hanger; 5, climbing and reaching the safe zone. The test was performed twice and each trial lasted a maximum of 3 min.

Motor deficits of 6-OHDA and A53T mutant α-synuclein model rats were assessed as previously described with slight modifications [27]. Briefly, both hind limbs and one forelimb were firmly fixed in the two hands of the experimenter, and the rat was lowered over a treadmill moving at rate of 18 cm/s such that the body remained stationary while the free forelimb was allowed to spontaneously touch the moving treadmill track for 10 s. All experimental sessions were video recorded to count the number of adjusting steps taken in the backward direction. The number of adjusting steps was averaged across four trials in each session.

In addition, impaired motor coordination of 6-OHDA model rats was examined using the rotarod test as previously described with slight modifications [28]. Tests were conducted prior to 6-OHDA injection (pre-6-OHDA), immediately after 6-OHDA injection (0d), after 7 days of KDS2010 administration (7d), and after 15 days of KDS2010 administration (15d). Before the first rotarod test, all rats were pre-trained until riding time reached 60 s at 10 rpm (B.S Technolab, Seoul, Korea). Each session consisted of two trials, and riding time was averaged to obtain pre-6-OHDA, 0d, 7d, and 15d results.

Motor dysfunction of A53T α-synuclein model rats was tested using the forelimb-use asymmetry test (cylinder test) [28]. Briefly, animals were placed in a transparent Plexiglas cylinder (20 cm in diameter and 30 cm in height) for 2 min to assess the frequency of ipsilesional and contralesional forelimb usage against the cylinder wall for supporting an upright body posture. The test score was calculated as the ratio of ipsilateral usage frequency to total ipsilateral plus contralateral usage frequency.

### Pharmacokinetics (PK) and Toxicity Study in Non-human Primates

All non-human primate studies were conducted in the Good Laboratory Practice (GLP)-level laboratory of the Korea Institute of Toxicology (KIT, Jeollabuk-do, Korea) and approved by the KIT Institutional Animal Care and Use Committee. Cynomolgus monkeys (*Macaca fascicularis*) were obtained from the Nafovanny captive-breeding primate facility (Dong Nai Province, Vietnam) and housed individually in stainless cages (543 W × 715L × 818H in mm) during acclimation, pre-treatment, and dosing periods. All animals were at least 24 months old. The environment of the animal room was automatically controlled according to the Standard Operating Procedures of KIT (temperature: 20–29°C, relative humidity: 30–70%, light cycle: 12 h at 300–700 Lux, ventilation: 10–20 times/h, air pressure: negative). Males and females were assigned to treatment groups in a stratified manner using the Pristima System based on most recent body weight. Clinical signs, including mortality, moribundity, general appearance, and behavioral changes, were recorded with date, time, and duration. Body weight was measured prior to the first KDS2010 dose. For oral administration, KDS2010 was dissolved in water. In total, 42 monkeys were administered KDS2010 and ultimately sacrificed for autopsy. The four studies conducted on cynomolgus monkeys are summarized in Table 1 and described in greater detail below.

**Table 1:** Overview of non-human primates (cynomolgus monkey) studies

Study no	Animals per group Main/recovery	Duration of dosing	Doses (mg/kg/day)	Terminal sacrifice day	
N118029	3 males/-	1 time	10 *p.o*	-	PK
	3 males/-		30 *p.o*		
N217019	1 male/- 1 female/-	1 time in 1st week	25 *p.o*	22	Single-dose escalation
		1 time in 2nd week	50 *p.o*		
		1 time in 3rd week	100 *p.o*		
G217013	4 males/- 4 females/-	Once a day for 2 weeks	0, 25, 50, 100 *p.o*	15	Dose range-finding
G218027	3 males/1 male 3 females/1 female	Once a day for 4 weeks 2 weeks for recovery	0, 10, 20, 40 *p.o*	22/36	Repeated toxicity for 4 weeks Recovery for 2 weeks Toxicokinetics

In the pharmacokinetic study (**N118029**), six male monkeys were assigned to 2 groups receiving either 10 or 30 mg/kg oral KDS2010. Approximately 0.5 mL of blood was collected from the cephalic vein pre-dose (time 0) and 0.5, 1, 2, 4, 6, 8, 10, and 24 h post-dose in tubes containing anti-coagulant (EDTA-2 K). Blood samples were mixed gently, stored in a wet-ice cryo-rack, and centrifuged (approximately 13,200 rpm, 5 min, 4°C) to obtain plasma. The separated plasma was aliquoted into polypropylene tubes and stored frozen until analysis.

In the single-dose escalation study (**N118029**), each male and female monkey was administered oral KDS2010 at increasing doses of 25, 50, and 100 mg/kg/day once a week. Mortality and general symptoms were observed during the trial period. Body weight was also measured. Blood was collected for hematology, coagulation, and clinical chemistry tests pre-dose (0) and 8, 15, and 22 days post-dose. After administration of 100 mg/kg/day on day 22, an autopsy was performed to observe individual macroscopic findings.

In the repeated 2-week oral administration toxicity study (**G217013**) for 4-week dose range finding (DRF), monkeys were assigned to 4 groups of one male and one female. Each group was orally administered KDS2010 at 0, 25, 50, or 100 mg/kg/day once a day for 2 weeks. During the test period, general symptoms were monitored, weight and feed intake measured, and ophthalmological tests performed. Clinical pathological tests (hematology, coagulation, blood biochemistry, and urine tests) were also conducted. All animals were autopsied, during which individual macroscopic findings were recorded, organ weights measured, and samples obtained for histopathology.

In the repeated 4-week oral administration toxicity with 2-week recovery and toxicokinetic test (**G218027**), monkeys were assigned to 4 groups of 3 males and 3 females. Each group was orally administered KDS2010 at 0, 10, 20, or 40 mg/kg/day once a day for 4 weeks. Toxicity tests were conducted as described for study G217013. For toxicokinetic measurements, blood samples were collected pre-dose (time 0) and at 1, 2, 4, 6, 8, 10, and 24 h after oral administration on the first day (day 1) and the last day (week 4; day 28) of dosing.

A non-compartmental analysis module in Phoenix® WinNonlin® (version 6.4) (Certara Inc., CA, USA) was used to calculate pharmacokinetic (PK) parameters. Systemic exposure to KDS2010 was calculated by applying the linear trapezoidal rule to the area under the plasma concentration–time curve from time zero to the last quantifiable time point (*AUC*_last_), and the maximum observed peak plasma concentration (*C*_max_) and the time to reach *C*_max_ (*T*_max_) were determined based on the observed data. The apparent terminal elimination half-life (*t*_1/2_) was calculated from the apparent terminal elimination rate constant using the formula *t*_1__/2_ = 0.693/kel.

### Data Presentation and Statistical Analysis

Studies were designed to generate groups of equal size. Statistical analyses were applied only if group size (*n*) ≥ 5. At least three independent repeats were performed for each experiment except for western blotting. Statistical analyses were not performed for TH staining of A53T mutant synuclein model rats at 3 weeks or for western blotting results because these experiments were design a priori as exploratory. Group size was chosen based on the expected difference from pilot studies in mice using similar protocols without conducting formal power analysis. Outliers were not excluded from data analysis and presentation, so group size is also the number of independent values. Except for Fig. 6 c, d, i, and f, all graphs present raw values. For Fig. 6 c, d, i, and f, we calculated the ratio of values for the ipsilateral and contralateral sides because the rats were unilaterally lesioned hemi-parkinsonian models.

All statistical analyses were conducted using Prism 8 (GraphPad). Differences between two groups were analyzed by two-tailed Student’s unpaired *t*-test. Within-group changes following a given intervention were assessed by two-tailed Student’s paired *t*-test. Multiple group means were compared by one-way analysis of variance (ANOVA) with Tukey’s multiple comparison tests or two-way ANOVA with Bonferroni’s multiple comparison tests as indicated. Data was assumed to be normally distributed. Brown-Forsythe and Welch one-way ANOVA with post hoc Dunnett T3 tests were conducted when the *F* values reached *P* > 0.05 and there was no significant variance inhomogeneity. A *P* < 0.05 was considered statistically significant for all tests. Significance level is represented by the number of asterisks (*P < 0.05, **P < 0.01, ***P < 0.001; ns, not significant). Unless otherwise specified, all data are presented as mean ± standard error of the mean (SEM).

## Results

### KDS2010 Is the Most Potent and Selective Monoamine Oxidase-B Inhibitor Among α-Amino Amide Derivatives

**Fig. 1:**
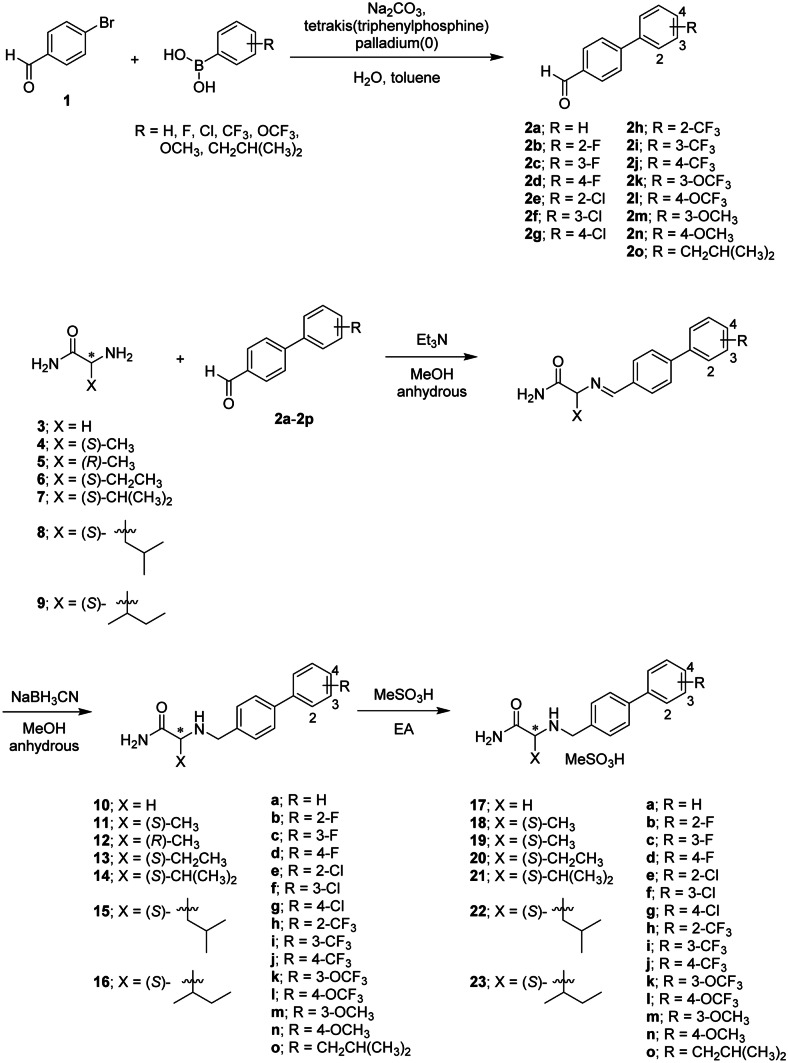
Synthetic scheme for α-aminoamide derivatives **17**–**23**

In our previous study, we synthesized a series of α-aminoamide derivatives by introducing biphenyl groups with various substituents as potential novel MAO-B inhibitors [14] and demonstrated that introduction of an electron-withdrawing group at the para-position is key to potent MAO-B inhibition. In the current study, we designed and synthesized additional derivatives by diversifying the alkyl group of α-aminoamide compounds (Fig. 1). The synthetic pathways for these α-aminoamides (compounds **17**–**23**) containing both different alkyl groups at the X position and various functional groups on the biphenyl ring B are illustrated in Fig. 1.

We first replaced the X position methyl group introduced previously [14] with a hydrogen and compared the inhibitory effects on MAO-B (Table 2). However, substitution of the CH_3_ group with H in compound **18j** (KDS2010), yielding compound **17j**, resulted in a 15-fold increase in the IC_50_ for MAO-B inhibition (from 8 to 121 nM). Next, we introduced a bulky alkyl group, such as ethyl, isopropyl, isobutyl, and sec-butyl, to the X position, and found that the inhibitory effect decreased dramatically with increasing alkyl group size. As in the previous study, the compounds with electron-withdrawing groups introduced at the para-position of biphenyl ring B increased MAO-B inhibition in the rank order CF_3_ > OCF_3_ > Cl > F. In contrast, addition of electron-donating groups (OCH_3_ and CH_2_CH(CH_3_)_2_) significantly reduced the inhibitory efficacy. Overall, **18j** (KDS2010) showed the strongest inhibitory effect on MAO-B among synthesized α-aminoamide derivatives, and much greater selectivity for MAO-B than the clinical MAO-B inhibitors selegiline and sembragiline. Furthermore, the (*S*)-stereoisomer (**18j**) of KDS2010 exhibited an eightfold higher inhibitory effect than the (*R*)-isomer (**19j**). We also found that **18j** is a competitive inhibitor with the same binding cavity as selegiline, and is reversible with enantiomeric selectivity [14]. Therefore, we selected KDS2010 (**18j**) for an in-depth evaluation of in vivo therapeutic efficacy in mouse and rat PD models. Further, we investigated its PK and toxicity profile in non-human primates to provide additional support for KDS2010 as a potential clinical drug candidate.

**Table 2:**
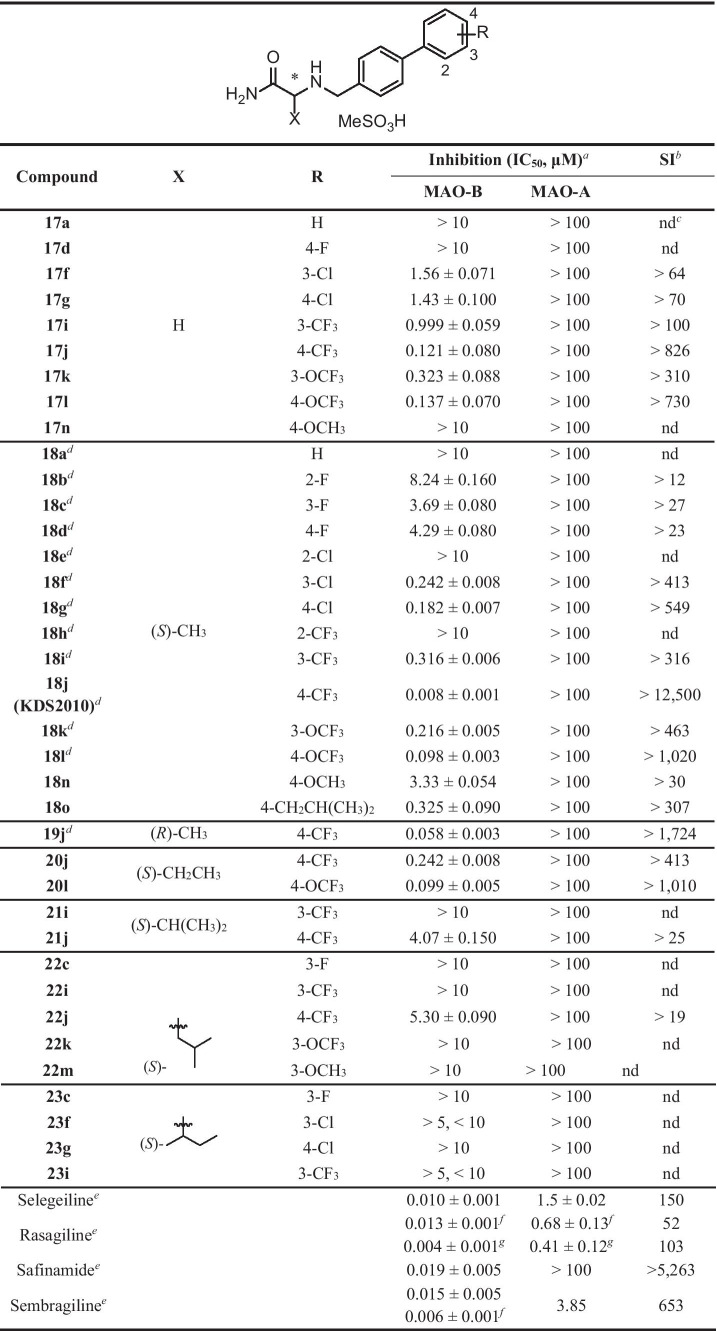
Inhibitory effects of α-aminoamide derivatives on hMAO enzymes

^a^The inhibitory capacity of MAOs is expressed in IC_50_ (μM) and the SEM is less than ± 1%

^b^Selectivity index (SI) provides selectivity for the MAO-B isoform at the ratio of IC_50_(MAO-A)/IC_50_(MAO-B)

^c^*nd*, not determined

^d^Park, JH. et al. Newly developed reversible MAO-B inhibitor circumvents the shortcomings of irreversible inhibitors in Alzheimer’s disease. *Science Advances*. 5(3), eaav0136 (2019)

^e^Positive controls; selegiline and rasagiline: irreversible inhibitor, safinamide, and sembragiline: reversible inhibitor

^f^Borroni, E. et al. Sembragiline: a novel, selective monoamine oxidase type B inhibitor for the treatment of Alzheimer’s disease. *J. Pharmacol. Exp. Ther*. 362, 413–423 (2017)

^g^Moussa B.H. Y. et al. Rasagiline [*N*-propargyl-1*R*( +)-aminoindan], a selective and potent inhibitor of mitochondrial monoamine oxidase B. *Br. J. Pharmacol.* 132, 500–506 (2001)

### Short-Term KDS2010 Treatment Alleviates Parkinsonism

**Fig. 2:**
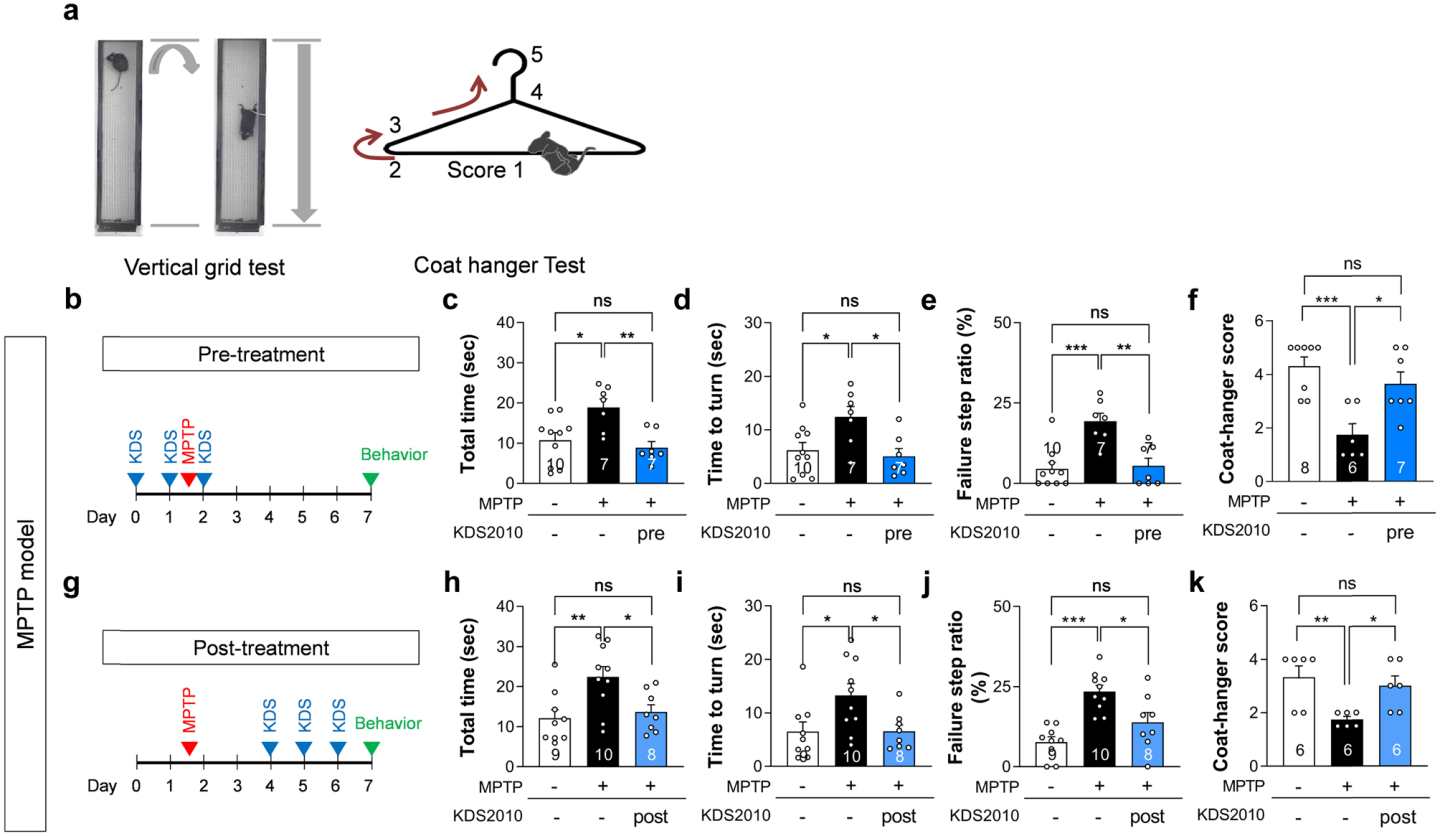
Pre- and post-treatments with KDS2010 alleviate parkinsonian motor deficits in MPTP model. **a** Schematic diagram of vertical grid test and coat hanger test. **b** Timeline of pre-treatment with KDS2010. **c**–**e** Total time, time to turn, and failure step ratio assessed by vertical grid test. Pre-treatment with KDS2010 reduced total time, time to turn, and failure step ratio in MPTP-treated animals. **f** Motor score assessed by coat hanger test. Pre-treatment with KDS2010 rescued the score in MPTP-treated animals. **g** Timeline of post-treatment with KDS2010. **h**–**j** Total time, time to turn, and failure step ratio assessed by vertical grid test. Post-treatment with KDS2010 reduced total time, time to turn, and failure step ratio in MPTP-treated animals. **k** Motor score assessed by coat hanger test. Post-treatment with KDS2010 rescued the score in MPTP-treated animals

To test the in vivo efficacy of KDS2010 against PD-like pathology, we first examined the effects of oral dosing against neurological and behavioral abnormalities induced by the selective dopaminergic neurotoxin 1-methyl-4-phenyl-1,2,3,5-tetrahydropyridine (MPTP), among the most widely studied animal models of PD [18]. In vivo, MPTP is converted to MPP+ through the enzymatic action of MAO-B in astrocytes and selectively transported into dopaminergic neurons via a monoamine transporter where it interferes with mitochondrial respiration [29]. Therefore, the therapeutic actions of MAO-B inhibitors in the MPTP model are believed to be mediated by blockade of MPTP to MPP+ conversion in astrocytes [30]. As most previous studies treated model animals with MAO-B inhibitors before MPTP administration [8, 31, 32], we treated the animals with oral KDS2010 (10 mg/kg/day) for 3 consecutive days starting 1 day before MPTP administration (pre-treatment; Fig. 2b). First, to test if KDS2010 pre-treatment alleviates MPTP-induced parkinsonian motor symptoms, mice were examined in the vertical grid test (Fig. 2a) [23]. Pre-treatment with KDS2010 significantly reduced the total time, time to turn, and rate of missed steps (Fig. 2c–e). Further, KDS2010 significantly improved motor coordination and balance as evaluated by the coat hanger climbing test [33, 34] (Fig. 2a) compared to vehicle-treated controls (motor function score: 3.6 ± 0.5 vs. 1.8 ± 0.4). (Fig. 2f).

If the therapeutic effects of KDS2010 on MPTP-induced PD-like pathology are mediated solely by blockade of MPTP conversion into MPP+ , KDS2010 may not be an effective clinical drug as MPTP-induced PD is rarely seen. Therefore, we examined the therapeutic effects of KDS2010 independent of MPTP to MPP+ conversion by delaying administration until 3 days after MPTP treatment (post-treatment; Fig. 2g). Previous studies have clearly demonstrated that striatal MPP+ concentration reaches a peak within 1 to 2 h after *i.p.* or *s.c.* MPTP administration but is detected for only 12 to 24 h thereafter [35, 36], suggesting that any effects after a 3-day delay in KDS2010 treatment would be independent of MPTP to MPP+ conversion. Even when administered 3 days after MPTP, however, KDS2010 still significantly reduced the total time, time to turn, and rate of missed steps in the vertical grid test (Fig. 2h–j) and increased motor score in the coat hanger test (Fig. 2k) compared to vehicle-treated controls. Selegiline also showed significant therapeutic efficacy in the MPTP model when administered either as post-treatment or pre-treatment [8]. These results indicate that the therapeutic efficacy of KDS2010 against MPTP toxicity involves actions unrelated to blockade of MPTP to MPP+ conversion.

### Short-Term KDS2010 Treatment Alleviates Nigrostriatal Tyrosine Hydroxylase Loss and Neuroinflammation

**Fig. 3:**
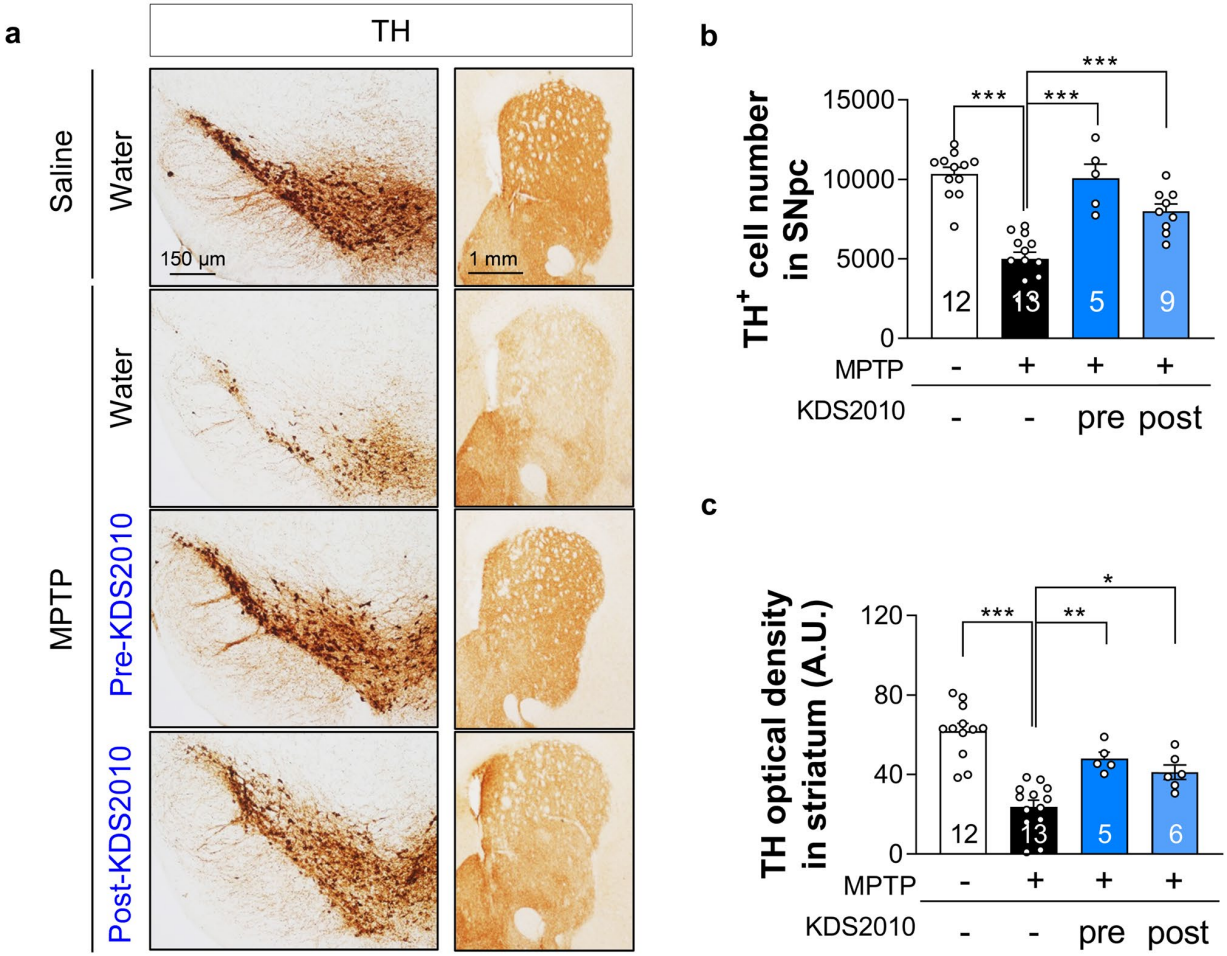
Pre-treatment and post-treatment with KDS2010 rescue nigrostriatal TH loss in MPTP model. **a** Representative images of TH-stained SNpc and striatum tissues. **b** Quantification of TH-positive cell number in SNpc. Both pre-treatment and post-treatment with KDS2010 significantly rescued the number of TH-positive dopaminergic neurons in SNpc of MPTP-treated animals. **c** Quantification of TH optical density in striatum. Both pre-treatment and post-treatment with KDS2010 significantly mitigate the TH loss in striatum of MPTP-treated animals

To examine if alleviation of MTPT-induced motor dysfunction by KDS2010 is associated with protection of nigrostriatal dopaminergic neurons, we first assessed the nigrostriatal expression level of TH, the rate-limiting enzyme in dopamine biosynthesis. As expected, MPTP administration alone significantly reduced TH-positive cell number in the SNpc as well as TH optical density in the striatum (Fig. 3), while both KDS2010 pre-treatment and post-treatment significantly increased TH-positive cell number in the SNpc and TH optical density in the striatum compared to MPTP plus vehicle (Fig. 3). These findings suggest that the alleviation of motor dysfunction by KDS2010 is associated with preservation of nigrostriatal dopaminergic neuron function or viability.

To directly confirm KDS2010-mediated neuroprotection, we then examined if KDS2010 treatment enhanced the number of SNpc neurons showing Nissl staining, a histological indicator of active protein synthesis by viable cells [37]. Again as expected, MPTP administration reduced the number of Nissl-positive neurons in the SNpc (Supplementary Fig. 1). This reduction was much less severe than the reduction in TH-positive neurons (Fig. 3b and Supplementary Fig. 1), suggesting the possible presence of TH-negative live neurons in the SNpc as we previously demonstrated [8]. Pre-treatment but not post-treatment with KDS2010 partially prevented the MPTP-induced reduction in Nissl-positive neurons (Supplementary Fig. 1). These findings suggest that KDS2010 pre-treatment protects dopaminergic neurons against MPTP-induced cell death, while post-treatment restores the dopamine transmission by rescuing the TH expression in TH-negative dormant neurons.

**Fig. 4:**
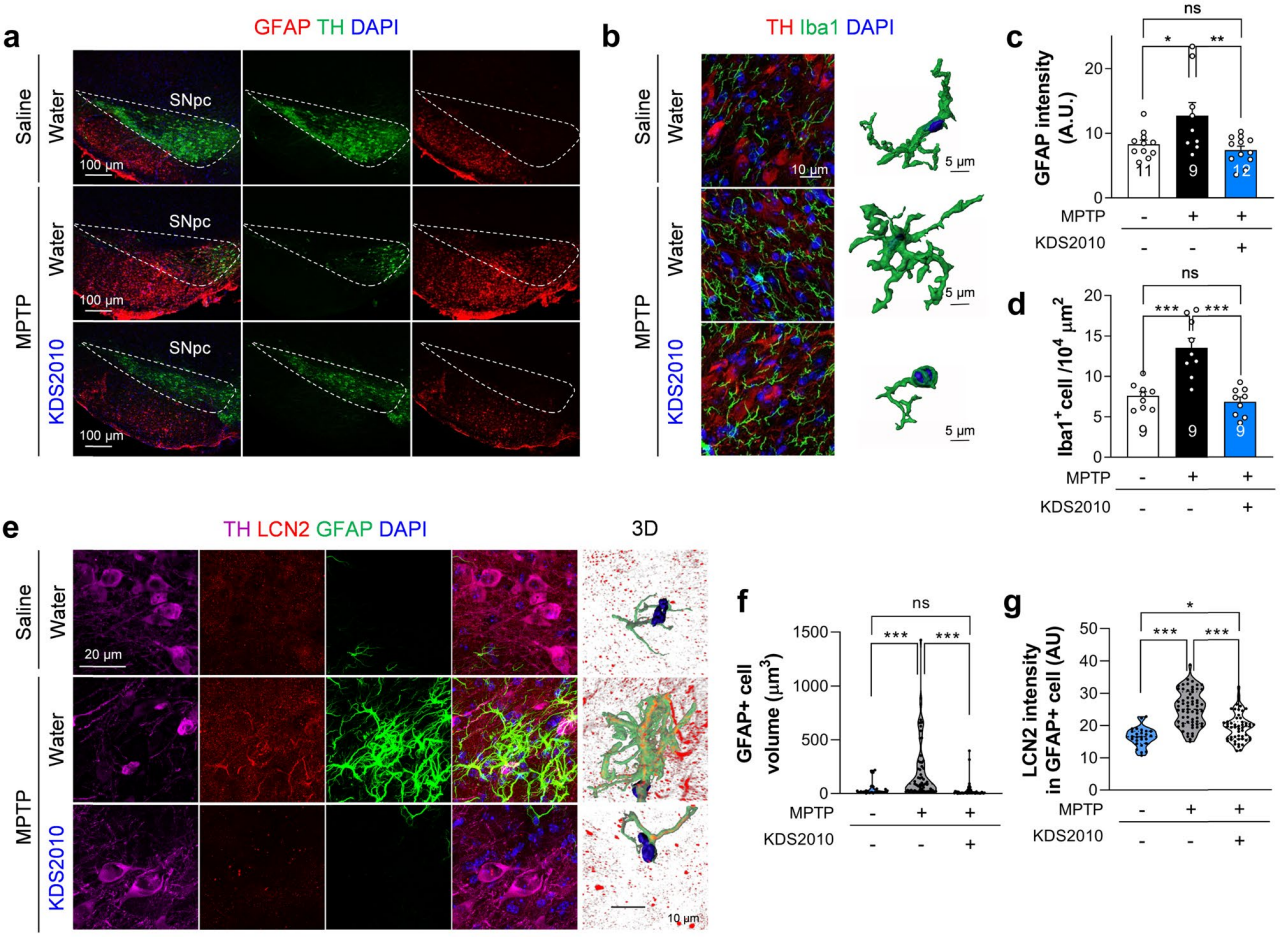
KDS2010 treatment alleviates glial reactivity. **a** Representative confocal images of GFAP and TH-stained SNpc tissues. **b** Representative images of TH and Iba1-stained SNpc tissues. **c** Quantification of GFAP intensity in SNpc area. MPTP significantly increased the GFAP intensity which was reversed by KDS2010 treatment. **d** Quantification of Iba1 intensity in SNpc area. MPTP significantly increased the Iba1 intensity which was reversed by KDS2010 treatment. **e** Representative confocal images of GFAP, LCN2, and TH-stained SNpc tissues. Right panel demonstrates 3D-rendered representative astrocytes. **f** Quantification of the volume of GFAP-positive cells. MPTP significantly increased the volume of GFAP-positive cells which was reversed by KDS2010 treatment. **g** Quantification of LCN2 intensity in GFAP-positive cells. MPTP significantly increased the LCN2 intensity which was reversed by KDS2010 treatment

Neuroinflammation is a major pathogenic pathway in PD [38]. Indeed, reactive astrocyte and microglial numbers are increased in the brains of PD model animals, including MPTP model animals, as well as in the brains of PD patients [8, 39]. We previously reported that this increased astrocytic reactivity is mediated by MAO-B in both PD and AD models [8, 14], and that KDS2010 treatment can dramatically reduce astrocytic reactivity in AD model animals [14]. Therefore, we tested if KDS2010 treatment also reduces astrocytic as well as microglial reactivity in the MPTP model by immunohistochemical staining for the reactive astrocyte markers GFAP and lipocalin 2 (LCN2), and for the reactive microglial marker Iba1. Consistent with induction of neuroinflammation, MPTP significantly increased both GFAP staining intensity and the number of Iba1-positive microglia, while KDS2010 significantly reduced these signs of neuroinflammation (Fig. 4a–d). In addition, many microglia exhibited morphological features of activation such as larger cell bodies and shorter processes following MPTP treatment, and this reactive transformation was reversed by KDS2010 treatment (Fig. 4b). The volume of GFAP-positive astrocytes was also increased in the SNpc of MPTP-treated mice, and this hypertrophy was eliminated by KDS2010 treatment (Fig. 4e, f). The expression of LCN2 in GFAP-positive astrocytes was also enhanced by MPTP, while KDS2010 reversed this response (Fig. 4e, g). Furthermore, western blotting demonstrated decreased TH expression and increased GFAP and inducible nitric oxide synthase (iNOS) expression following MPTP, again consistent with neuroinflammation, and all of these changes were reversed by KDS2010 treatment (Supplementary Fig. 2). These results are consistent with our previous findings in AD model mice [14] and indicate that KDS2010 is effective at reducing neuroinflammation, which is a major cause of dopaminergic neuron dysfunction and degeneration in PD.

### Long-Term KDS2010 Treatment also Mitigates the Parkinson’s Disease-like Pathology and Symptoms Induced by 1-Methyl-4-phenyl-1,2,3,6-tetrahydropyridine

**Fig. 5:**
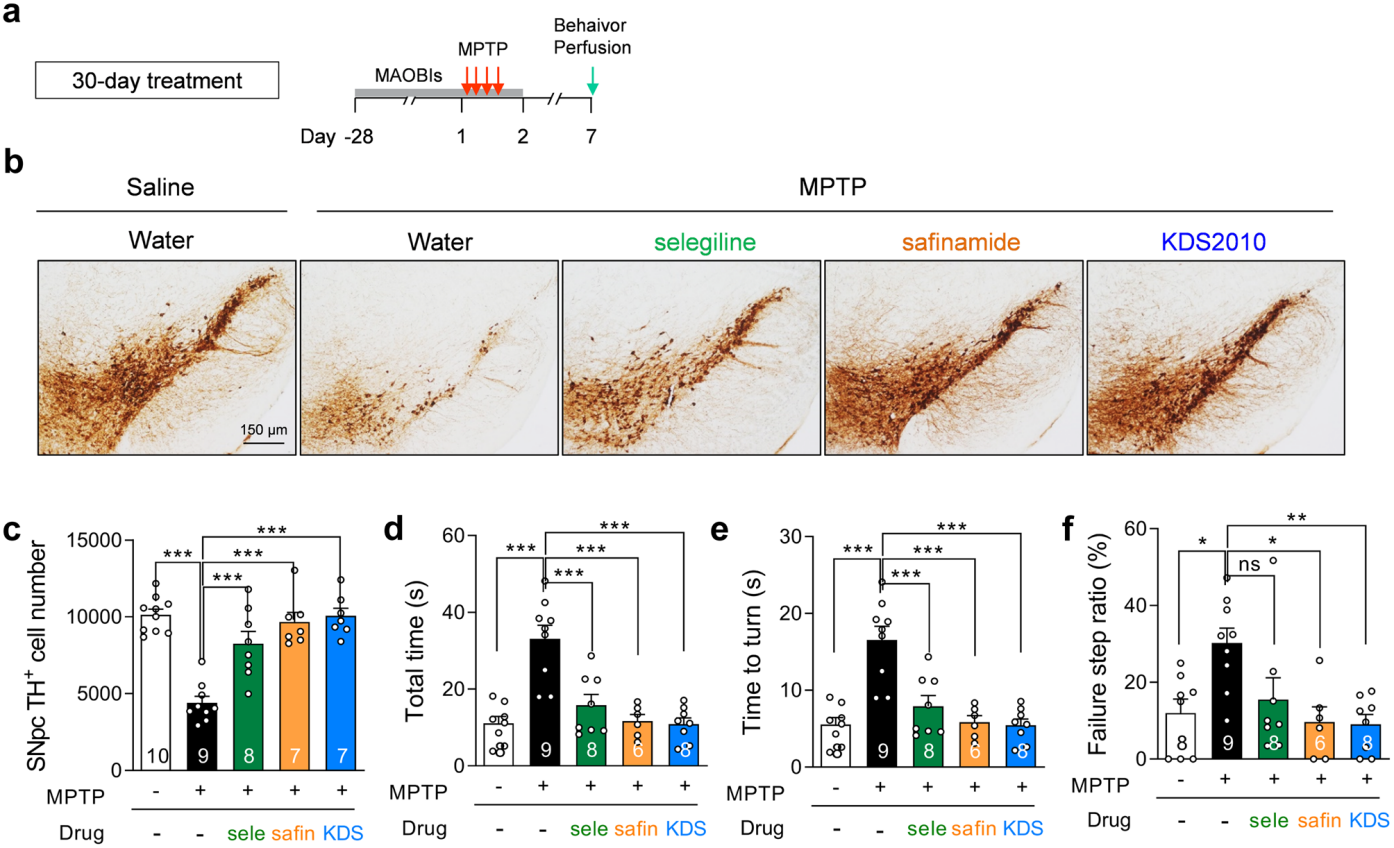
Long-term treatment of reversible MAO-B inhibitors significantly alleviates TH loss and parkinsonian motor deficits in MPTP model. **a** Timeline of long-term (30-day) treatment with MAO-B inhibitors, including selegiline, safinamide, and KDS2010. **b** Representative images of TH-stained SNpc tissues. **c** Quantification of TH-positive cell number in SNpc. Long-term treatment with safinamide and KDS2010 significantly rescued the number of TH-positive dopaminergic neurons in SNpc of MPTP-treated animals, while selegiline only showed a non-significant increasing tendency. **d**–**f** Total time, time to turn, and failure step ratio assessed by vertical grid test. Long-term treatment with safinamide and KDS2010 significantly rescued all items assessed in MPTP-treated animals, while selegiline only showed a non-significant reducing trend in failure step ratio

We next tested the long-term therapeutic efficacy of KDS2010 compared to the clinical MAO-B inhibitors selegiline and safinamide in MPTP model mice. Treatment with KDS2010, selegiline, or safinamide for 29 days before MPTP injection and for one more day after injection (Fig. 5a) significantly mitigated the loss of TH-positive cells in the SNpc (Fig. 5b, c) and reduced both total time and time to turn in the vertical grid test (Fig. 5d, e). However, only the reversible inhibitors KDS2010 and safinamide also significantly reduced the failure step ratio, whereas selegiline showed a non-significant partial efficacy (Fig. 5f). The partial but actual efficacy of selegiline may be explained by long-term treatment when the action of MAO-B-derived astrocytic GABA is minimal. At this time, compensatory astrocytic GABA synthesis through diamine oxidase (DAO) [14] may not alter behavioral and histological outcomes. This is in contrast to the finding that 15-day selegiline treatment was not effective for behavioral recovery following 6-OHDA treatment, when the pathological action of MAO-B-mediated astrocytic GABA production is critical (Supplementary Fig. 3c). These results suggest that long-term administration of reversible MAO-B inhibitors including KDS2010 may also mitigate PD pathology and symptoms in patients with aberrant astrocytic GABA synthesis.

### KDS2010 Is also Effective in Other Animal Models of Parkinson’s Disease

**Fig. 6:**
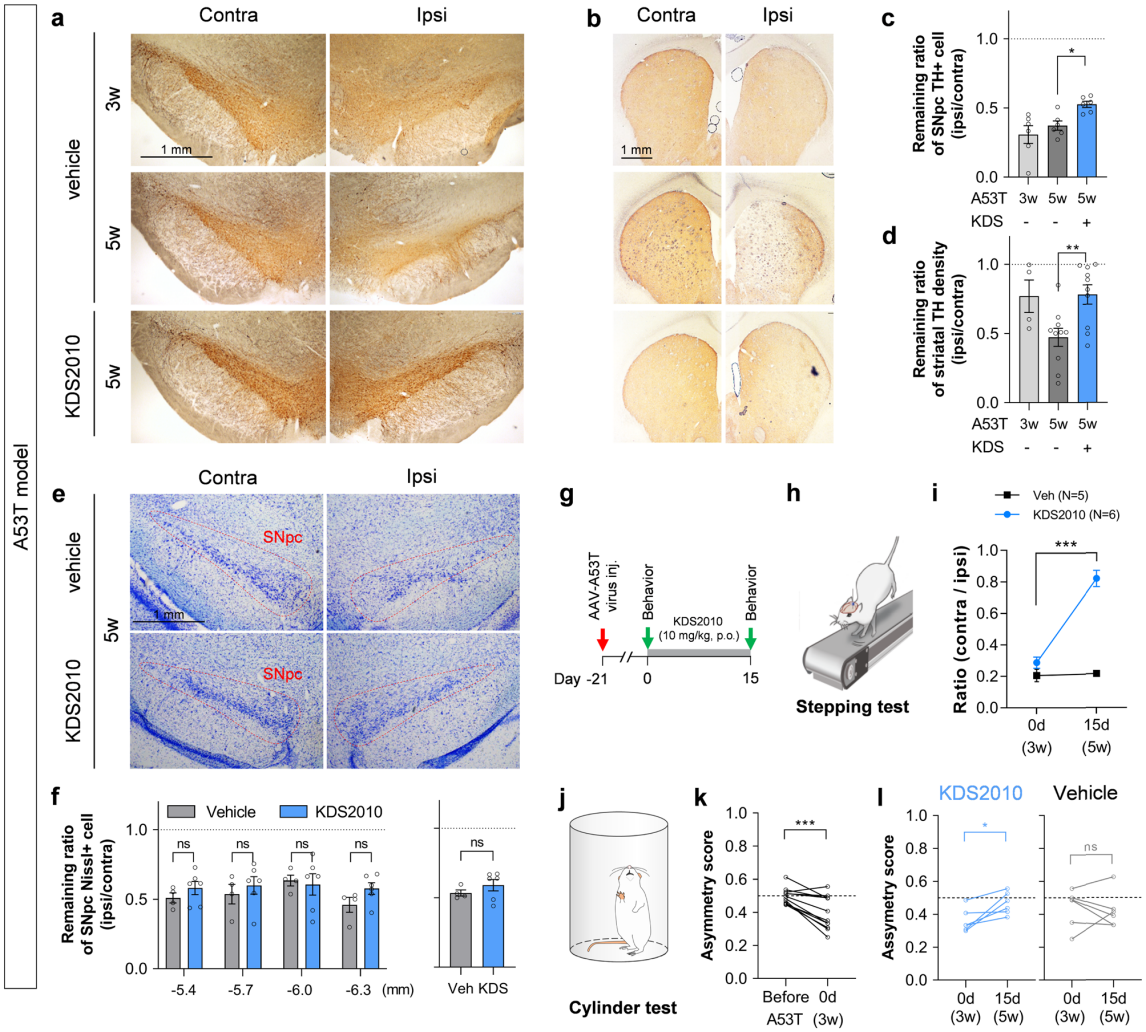
Treatment with KDS2010 alleviates parkinsonian motor deficits in A53T model. **a** Representative images of TH-stained SNpc slices. **b** Representative images of TH-stained striatal slices. **c** Quantification of remaining ratio of TH optical density in ipsilateral striatum. Both 3 and 5 weeks after A53T virus injection, remaining SNpc TH was marked reduced. KDS2010 treatment significantly rescued from the TH loss. **d** Quantification of remaining ratio of TH optical density in ipsilateral striatum. Five weeks after A53T virus injection, but not yet 3 weeks, remaining striatal TH was marked reduced. KDS2010 treatment rescued from the TH loss. **e** Representative images of Nissl staining. **f** Quantification of remaining ratio of Nissl-positive neurons in SNpc of the coronal brain sections at the AP level of -5.4, -5.7, -6.0, and -6.3 mm from the bregma. **g** Timeline of post-treatment with KDS2010 for 15 days in A53T model. **h** Schematic diagram of stepping test. **i** Quantification of the ratio of contralateral stepping numbers over ipsilateral ones. KDS2010 treatment significantly recovered the stepping ratio. **j** Schematic diagram of cylinder test. **k** Quantification of asymmetry score before and 3 weeks after A53T virus injection. **l** Quantification of asymmetry score before and after 15-day KDS2010 treatment starting from 3 weeks after A53T virus injection

Next, we tested the possible therapeutic efficacy of KDS2010 in A53T mutant α-synuclein-overexpressing PD model rats (A53T model) [20] established by AAV-A53T virus injection into the unilateral SNpc [8]. Unlike the toxin-based MPTP and 6-OHDA models, this PD model may replicate an endogenous pathomechanism of human PD. Three weeks after virus injection, ~ 70% of SNpc neurons on the injection side had already lost TH expression, whereas striatal TH levels were less affected (Fig. 6a–d), indicating that DA neuron dysfunction and degeneration were still ongoing, while at 5 weeks after virus injection, TH loss was evident in both SNpc and striatum, indicating that neurodegeneration had stabilized. Therefore, KDS2010 (10 mg/kg/day) administration was initiated for 15 days starting 3 weeks after virus injection when a fraction of dopaminergic neurons were presumably salvageable (Fig. 6g). Indeed, this 15-day KDS2010 administration significantly reversed striatal TH loss and increased the number of remaining TH-positive neurons in the injected SNpc compared to rats receiving virus injection alone (Fig. 6a–d). In addition to reduced TH staining, A53T α-synuclein overexpression caused a ~ 50% loss of Nissl-positive neurons (Fig. 6e–f). This was slightly less than the ~ 60% loss of TH-positive neurons, a discrepancy suggesting the possible presence of TH-negative live neurons as demonstrated in our previous work [8]. Our previous work further demonstrated the TH-negative (“dormant”) neurons in the SNpc of PD model mice can be induced to express TH by MAO-B blockade [8]. These findings strongly suggest that the KDS2010-mediated TH recovery is attributable at least in part to recovery of TH expression in dormant neurons.

We also assessed the effects of KDS2010 on motor behaviors of A53T mutant α-synuclein-overexpressing rats using the well-validated stepping test [27] (Fig. 6h) and found that KDS2010 treatment significantly alleviated the impaired stepping behavior of the contralateral forepaw observed in A53T model rats (Fig. 6i). We also performed the cylinder test to confirm KDS2010 efficacy against motor dysfunction in this A53T model (Fig. 6j). Model rats showed significant asymmetric usage of forelimbs (Fig. 6k), a deficit alleviated by KDS2010 but not vehicle treatment (Fig. 6l). This effect of KDS2010 may be attributed to protection of degenerating neurons overexpressing mutant α-synuclein because KDS2010 treatment was started when TH neurons were still recoverable. Taken together, these findings indicate that KDS2010 is highly effective for treating parkinsonian motor symptoms regardless of etiology.

Finally, we also tested the therapeutic efficacy of KDS2010 in 6-OHDA model rats, which exhibit more extensive DA neurodegeneration and severe motor deficits. Further, we pre-screened these animals using the apomorphine-induced rotation test. Animals were treated with KDS2010 (10 mg/kg/day) for 15 days starting 17 days post-6-OHDA injection, at which time DA neuronal loss is reported to be almost stabilized [22] (Supplementary Fig. 3), and motor function was assessed by the stepping test (Supplementary Fig. 3

). Treatment with KDS2010 partially but significantly improved the stepping behavior deficit of the contralateral forepaw induced by 6-OHDA (Supplementary Fig. 3c). On the contrary, 15 days of selegiline treatment did not improve stepping behavior (Supplementary Fig. 3c), which may be attributed to compensatory astrocytic GABA synthesis after long-term treatment with this irreversible MAO-B inhibitor [8]. We also performed the rotarod test to confirm the therapeutic efficacy of KDS2010 against 6-OHDA-induced motor dysfunction (Supplementary Fig. 3d) and found that 15 days’ treatment (also starting 17 days after 6-OHDA injection) partially reversed the reduction in riding time (i.e., increased the latency to fall) observed in these parkinsonism model animals (Supplementary Fig. 3e). On the other hand, nigrostriatal TH loss was not reversed by KDS2010 treatment (data not shown), possibly due to extensive loss of DA neurons before treatment onset. The therapeutic efficacy of KDS2010 may therefore be attributed to activation of a subpopulation of surviving DA neurons or to activation of compensatory motor circuits, alternatives that must be examined in future studies.

### KDS2010 Shows a Favorable Pharmacokinetic Profile and No Toxicity in Non-human Primates

To select an effective and safe dose for a first in human (FIH) study, we performed pharmacokinetic (PK) and toxicity testing in cynomolgus monkeys based on preclinical PK data from rats [14] . A single oral administration of 10 mg/kg KDS2010 to male monkeys yielded a serum *C*_max_ value of 2232.0 ± 142.7 ng/mL while 30 mg/kg yielded a *C*_max_ value of 4845.1 ± 567.5 ng/mL (Table 3). The *C*_max_ at 10 mg/kg was 2.34-fold higher in monkeys than rats (952.1 ± 80.3 ng/mL) (Table 3). In addition, the total blood concentration (*AUC*_all_) was 6 times higher in cynomolgus monkeys than rats following the same 10 mg/kg dose, indicating that oral administration results in superior drug exposure in primates compared to rodents (Table 3).

**Table 3:**
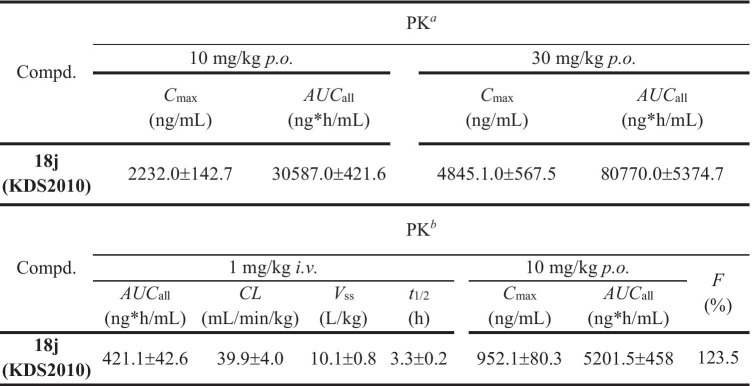
In vivo pharmacokinetic parameters of KDS2010

*Compd* compound; *AUC* area under the plasma concentration–time curve; *CL* time-averaged total body clearance; *V*_ss_ apparent volume of distribution at steady state; *t*_1/2_ elimination half-life; *C*_max_ maximum concentration of the drug; *F* bioavailability

^a^Monkeys (*n* = 3) were dosed with 10 mg/kg for *p.o.* or 30 mg/kg for *p.o.* Parameters were calculated from composite mean plasma concentration–time data. Data are expressed as the mean ± SD. %

^b^Rats (*n* = 5) were dosed with 1 mg/kg for *i.v.* and 10 mg/kg for *p.o.* Parameters were calculated from composite mean plasma concentration–time data. Data are expressed as the mean ± SD. %. From Park, JH. et al. Newly developed reversible MAO-B inhibitor circumvents the shortcomings of irreversible inhibitors in Alzheimer’s disease. *Science Advances*. 5(3), eaav0136 (2019)

**Fig. 7:**
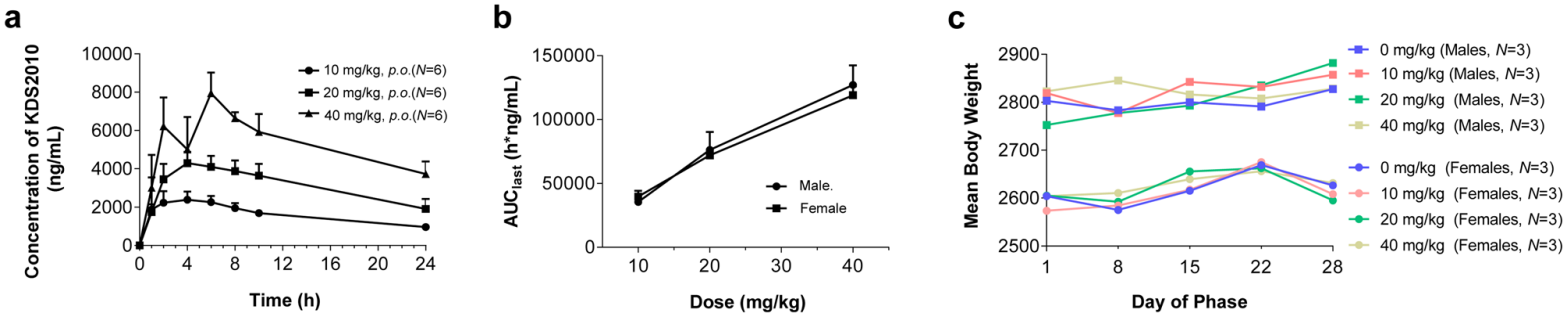
KDS2010 has a favorable PK profile in non-human primates and is safe for 4 weeks of repeated dosing. **a** Composite serum KDS2010 concentration–time profiles after single *p.o.* doses of 10, 20, and 40 mg/kg in cynomolgus monkey. **b** Increased amount of systemic exposure (*AUC*_last_) compared to dose. **c** Changes in body weight by group during the 4-week repeated dosing period

Next, we conducted single- and 4-week repeated-dose oral toxicity studies. In the single repeated-dose toxicity study (N217019), administration of 25, 50, and 100 mg/kg KDS2010 on successive days induced no general symptoms, clinical pathological, or gross organ pathology. For the 4-week repeated-dose oral toxicity study, we first conducted a 2-week DRF study (G217013) and determined the maximum dose to be 50 mg/kg/day, so KDS2010 was administered orally to male and female cynomolgus monkeys at 0, 10, 20, or 40 mg/kg/day, once a day, for 4 weeks. Toxicokinetic analysis revealed a mean T_max_ of 4.72 ± 1.93 h after administration (Fig. 7a) and a slightly less than proportional dose-dependent increase according to the *AUC*_last_ (Fig. 7b). During the test period, no significant changes in body weight were observed in male and female groups (Fig. 7c). Moreover, no adverse effects were observed in toxicity examinations (Supplementary Fig. 4). Taken together, KDS2010 has a No Observed Adverse Effect Level (NOAEL) of 40 mg/kg/day for both sexes. These results indicate high potential of KDS2010 as a clinical candidate drug for PD.

## Discussion

In this study, we demonstrate that KDS2010 is the most potent and selective MAO-B inhibitor among a large array of α-aminoamide derivatives. Moreover, a 3-day treatment with KDS2010 prevented or partially reversed MPTP-induced PD-like pathologies, including nigrostriatal TH loss, astrogliosis, microgliosis, and parkinsonian motor deficits. Further, long-term KDS2010 treatment in 6-OHDA model showed more favorable effect than selegiline, an irreversible MAO-B inhibitor widely prescribed for PD. We also demonstrated a favorable PK profile and low toxicity in non-human primates, validated KDS2010 as a potential therapeutic agent for PD patients.

### Advanced Pharmacological Properties of KDS2010 as a CNS Drug Candidate

In a previous study, we synthesized an array of α-aminoamide derivatives and found that the compound containing a biphenyl moiety, KDS2010, demonstrated highest selectivity for MAO-B inhibition over MAO-A inhibition and greatest inhibitory potency (lowest IC_50_) among these compounds as well as rapid reversibility [14]. This novel compound also exhibited the highest potency in both in vitro and in vivo rodent models, and with better absorption, distribution, metabolism, excretion, and toxicity (ADME/Tox) profiles than other α-aminoamide derivatives [14]. Moreover, repeated 4-week oral administration toxicity studies of cynomolgus monkeys indicated that KDS2010 is well tolerated and essentially non-toxic at daily doses up to 40 mg/kg. We also found that drug exposure after oral administration was significantly higher in primates than rodents, indicating that the appropriate dose for a FIH study should be based on pharmacokinetics in monkeys. Taken together, KDS2010 shows favorable PK parameters, high molecular target specificity, and good in vivo safety, critical preclinical requirements for CNS drug candidates [14].

### Possible Action Mechanisms of KDS2010 in Parkinson’s Disease

What are the possible mechanisms for these in vivo therapeutic effects of KDS2010? First, KDS2010 likely blocks the conversion of MPTP to 1-methyl-4-phenylpyridinium (MPP+) by MAO-B in astrocytes [29], which would explain the therapeutic effects of KDS2010 pre-treatment. On the other hand, several previous reports have clearly demonstrated that the conversion of MPTP to MPP+ and the clearance of MPP+ in the striatum are completed within 24 h after systemic MPTP administration. These reports served as the basis for our post-treatment experiments in which KDS2010 treatment was delayed until 72 h after MPTP injection. However, post-treatment of KDS2010 still dramatically reversed the MPTP-induced reduction in nigrostriatal TH expression and the emergence of parkinsonian motor symptoms, suggesting an alternative action other than blockade of MPTP to MPP+ conversion by MAO-B. One possibility is blockade of dopamine degradation. However, there are conflicting results on the contribution of MAO-B to dopamine degradation. For instance, no difference in dopamine level was found between MAO-B deficient mice and wild-type mice [35]. In addition, no alteration in dopamine level was found under selegiline treatment [40].

In addition to these two possibilities, we have reported a critical role for MAO-B in GABA synthesis by reactive astrocytes [8–11]. In a previous study, we found that astrocytic GABA level was aberrantly increased in the SNpc of PD model animals, that neighboring DA neurons were tonically inhibited, and that this inhibition was dependent on both MAO-B and the GABA_A_ receptor α5 subunit (GABA_A_Ra5) [8]. We also found that DA neuronal firing rate was reduced in the SNpc of PD model brain due to MAO-B-dependent GABA production [8]. This excessive tonic inhibition may cause some DA neurons to become dormant. In this state, dopaminergic neurons expressed relatively normal levels of DOPA decarboxylase (DDC) but relatively low levels of TH. In fact, the proportion of TH^l^°^w^/DDC^+^ neurons was reduced by pharmacological inhibition or genetic deletion of MAO-B. These findings suggest that GABA from reactive astrocytes contributes critically to DA neuronal dysfunction and parkinsonian motor symptoms. Based on this physiological evidence, we suggest that the benefits of KDS2010 in PD animal models may result from disinhibition of DA neurons via blockade of MAO-B-mediated GABA production.

Reactive astrocytes are reported to produce excessive amount of hydrogen peroxide concomitant with GABA synthesis via MAO-B [41]. Therefore, in addition to blockade of astrocytic GABA synthesis, the therapeutic effect of KDS2010 may be mediated by blockade of astrocytic hydrogen peroxide production and ensuing oxidative stress (although KDS2010 does not directly scavenge hydrogen peroxide, Supplementary Fig. 5). Collectively, these findings suggest that KDS2010 treatment could activate dormant DA neurons by reducing aberrant GABA-mediated inhibition, and decelerate DA neuronal death by reducing aberrant hydrogen peroxide accumulation, ultimately leading to alleviation of parkinsonian motor symptoms.

The potential clinical utility of KDS2010 is supported by its efficacy in multiple PD models. However, efficacy differed somewhat among these models. The partial effect of KDS2010 in the A53T and 6-OHDA models could be attributed to the timing of the administration. In both models, KDS2010 treatment was started 3 weeks after injection of AAV-A53T virus or 6-OHDA when the neurodegeneration was already substantial. Thus, early treatment onset may have resulted in a more significant neuroprotective effect, a possibility warranting further study.

A 15-day treatment with KDS2010 was also more effective than selegiline for alleviation of PD-like pathology in 6-OHDA model mice, suggesting that reversible MAO-B inhibitions are therapeutically favorable to irreversible inhibition. In an animal model of AD, long-term treatment with selegiline is known to cause a rebound in astrocytic GABA through a DAO-mediated compensatory mechanism, whereas KDS2010 may not induce this response [14]. This hypothesis also warrants further study in PD animal models.

### Therapeutic Potential of KDS2010 for Other Neurological Disorders

In addition to anti-parkinsonian potential, KDS2010 effectively ameliorated memory impairment in the APP/PS1 transgenic mouse model of AD regardless of whether treatment was short-term (3 days) or long-term (30 days). This effect was attributed to blockade of astrocytic GABA synthesis and ensuing reduction in tonic inhibition of hippocampal neurons [14]. In the same study, we also found that long-term selegiline treatment (> 2 weeks) had no therapeutic effect due to compensatory upregulation of astrocytic GABA synthesis via diamine oxidase, while long-term treatment with a reversible MAO-B inhibitor did not activate this compensatory mechanism [14]. Furthermore, in our recent study, long-term treatment with KDS2010 dramatically improved post-stroke recovery when accompanied by rehabilitation training [11].

#### Conclusion

We have demonstrated that MAO-B is a key molecular target for suppression of neuroinflammation in both PD and AD, suggesting that reversible MAO-B inhibitors with good biosafety and bioavailability are promising candidate treatments for these neurodegenerative disorders. Furthermore, astrogliosis could be an indication for reversible MAO-B inhibitor treatment. Among reversible MAO-B inhibitors, KDS2010 demonstrates unprecedented potency, specificity, and safety. We propose that KDS2010 may be a highly effective therapeutic candidate for PD as well as other neuroinflammatory brain disorders.

## Supplementary Information

Below is the link to the electronic supplementary material.

Supplementary file1 (DOCX 9305 KB)

Supplementary file2 (PDF 518 KB)

Supplementary file3 (PDF 536 KB)

Supplementary file4 (PDF 536 KB)

Supplementary file5 (PDF 517 KB)

Supplementary file6 (PDF 527 KB)

Supplementary file7 (PDF 527 KB)

Supplementary file8 (PDF 544 KB)

Supplementary file9 (PDF 43 KB)

Supplementary file10 (PDF 43 KB)

Supplementary file11 (PDF 43 KB)

Supplementary file12 (PDF 43 KB)

Supplementary file13 (PDF 43 KB)

Supplementary file14 (PDF 44 KB)

Supplementary file15 (PDF 49 KB)

Supplementary file16 (PDF 43 KB)

Supplementary file17 (PDF 43 KB)

Supplementary file18 (PDF 43 KB)

Supplementary file19 (PDF 43 KB)

Supplementary file20 (PDF 43 KB)

Supplementary file21 (PDF 527 KB)

Supplementary file22 (PDF 1544 KB)

## Data Availability

All data underpinning this study is available from the authors upon reasonable request.

## Abbreviations

MAO-B: Monoamine oxidase-B
PD: Parkinson’s disease
MPTP: 1-Methyl-4-phenyl-1,2,3,6-tetrahydropyridine
SNpc: Substantia nigra pars compacta
DA: Dopaminergic
AD: Alzheimer’s disease
IC_50_: 50% Inhibitory concentration of compounds
EGCG: Epigallocatechin gallate
6-OHDA: 6-Hydroxydopamine
BCA: Bicinchoninic acid
PBS: Phosphate-buffered saline
TH: Tyrosine hydroxylase
PK: Pharmacokinetic
GFAP: Glial fibrillary acidic protein
LCN2: Lipocalin 2
iNOS: Inducible nitric oxide synthase
DRF: Dose range finding
NOAEL: No Observed Adverse Effect Level
ADME/Tox: Absorption, distribution, metabolism, excretion, and toxicity
MPP+: 1-Methyl-4-phenylpyridinium
GABA_A_Ra5: GABA_A_ receptor α5 subunit
DDC: DOPA decarboxylase
