# Supplementary material for: KDS2010, a Newly Developed Reversible MAO-B Inhibitor, as an Effective Therapeutic Candidate for Parkinson’s Disease
Source: Neurotherapeutics. 2021 Oct 5;18(3):1729–47. doi: 10.1007/s13311-021-01097-4 (PMC8608967; doi:10.1007/s13311-021-01097-4)
Supplement: Supplementary file 1 — Supplementary file1 (DOCX 9305 KB) [file 13311_2021_1097_MOESM1_ESM.docx]

**Supplementary Figures**

**KDS2010, a newly developed reversible MAO-B inhibitor, as an effective therapeutic candidate for Parkinson’s disease**

Min-Ho Nam1†, Jong-Hyun Park2†, Hyo Jung Song2†, Ji Won Choi2, Siwon Kim2,3, Bo Ko Jang2, Hyung Ho Yoon4, Jun Young Heo1,5, Hyowon Lee1, Heeyoung An6,7, Hyeon Jeong Kim2,8, Sun Jun Park2,3, Doo-Wan Cho9, Young-Su Yang9, Su-Cheol Han9, Sangwook Kim10, Soo-Jin Oh1, Sang Ryong Jeon4, Ki Duk Park2,3*, C. Justin Lee1,6*

**Figure S1. Pre-treatment of KDS2010 protects the MPTP-mediated loss of Nissl-positive neurons in SNpc.**

(a) Representative images of Nissl staining of SNpc tissues of MPTP PD models. (b) Quantification of Nissl-positive neurons in SNpc at the diverse anterior-posterior coordinate levels. MPTP partially but significantly reduced the number of Nissl-positive neurons in the SNpc, and pre-treatment of KDS2010 protected it.

**Figure S2. KDS2010 alleviates MPTP-induced TH loss and increase of iNOS and GFAP.**

(a) Western blot of TH, iNOS, GFAP and beta-actin. (b-d) Quantification of expression levels of TH, iNOS, and GFAP normalized by actin. SNpc tissues of MPTP model showed a reduced expression of TH and increased expression of iNOS and GFAP. KDS2010 treatment alleviates these MPTP-induced alterations.

**Figure S3. KDS2010 alleviates 6-OHDA-induced parkinsonian motor deficits.**

(a) Experimental timeline with 6-OHDA rat model. (b) Schematic diagram of stepping test. (c) Quantification of the stepping ratio of contralateral over ipsilateral forepaw. KDS2010 treatment significantly restore the motor function, while selegiline treatment did not. (d) Schematic diagram of rotarod test. (e) Quantification of the latency-to-fall in the rotarod test. KDS2010 treatment partially but significantly increased the latency-to-fall.

**Figure S4. Additional toxicity tests for KDS2010 in non-human primates**

(a) Quantification of hematology values in 4-week repeated dose toxicity study (WBC, white blood cell; RBC, red blood cell; HGB, hemoglobin; HCT, hematocrit; MCV, mean corpuscular volume; MCH, mean corpuscular hemoglobin; MCHC, mean corpuscular hemoglobin concentration; PLT, platelet). (b) Quantification of coagulation values (PT, prothrombin time; APTT, activated partial thromboplastin time). (c) Quantification of the ratio of organ weights over the body weight. (d) Quantification of urinalysis values (SG, Urinary specific gravity).

**Figure S5. KDS2010 does not directly scavenge H2O2.**

KDS2010 did not show any H2O2 scavenging effect, while a well-known H2O2 scavenger, epigallocatechin-3-gallate (EGCG), showed a dose-dependent effect of H2O2 scavenging.
